# Observations on the Zinc Induced Testicular Teratomas of Fowl

**DOI:** 10.1038/bjc.1964.14

**Published:** 1964-03

**Authors:** J. Guthrie

## Abstract

**Images:**


### OBSERVATIONS ON THE ZINC INDUCED TESTICULAR TERATOMAS OF FOWL

J. GUTHRIE

*From the Department of Pathology, St. Mary's Hospital, London, W.2*

Received for publication January 25, 1964

THE induction of teratomas in the testes of fowl by the intra-testicular injection of zinc salts has three problems of current interest in oncology.

1. The relationship of the inducibility of the teratoma to the functional state of the testis and related endocrine organs and the possible hormone dependence of the growing tumour.
2. The mechanism of action of the metallic salt as a carcinogen.
3. The nature of any chromosomal differences from the parent tissue in view of the disputed origin of teratomas.

Initial observations on the third problem have been reported (Guthrie, 1962a) in a study of the chromosomal structure of experimental teratomas in fowl. The present report deals with aspects of the first two.

Spontaneous testicular teratomas appear to be very rare in birds (Mashar, 1932 ; Campbell, 1951).

The seasonal aspect of the inducibility of these testicular teratomas in fowl was noted by nearly all the earlier authors, both in Russia and America (Michalowsky, 1926, 1928 and 1929 ; Bagg, 1936 and 1937 ; Anissimowa, 1939 ; Falin and Gromzewa, 1939 and 1940 ; Falin and Anissimowa, 1940 ; Falin, 1940 and 1941 ; Carleton, Friedman and Bomze, 1953 ; Smith and Powell, 1957). They also gave detailed description of their histological structure. In general the teratomas could only be induced by intra-testicular inoculation of solutions of zinc chloride, sulphate or nitrate and copper sulphate during the months January to March. The age of the tumour bearers at the time of inoculation varied from 5 to 18 months, but in several of the previous reports the age of the fowl was either unknown or not stated. Tumours have been successfully induced in both light and heavy breeds and in hybrids. Carleton *et al.* (1953) found the highest incidence of tumours in White Leghorns inoculated at 18 months of age although they do not give particulars of their experience with other breeds. Bresler (1959 and 1962) reported testicular tumours including teratomas in mice following intra-testicular injections of copper sulphate solution and androgen administration, but apart from this the only experimental teratomas have been those induced in fowl. It must be remembered that Stevens and Little (1954) have reported a high incidence (1 per cent) of spontaneous testicular teratomas in strain 129 mice.

Table I summarises the previous work in chronological order.

### MATERIALS AND METHODS

### *Preparation of inocula*

*Zinc chloride.*—The solution was prepared as 5 g. zinc chloride/100 ml. distilled water B.P. and cloudiness due to precipitation of zinc hydroxide removed by adjusting the pH to 3·2 by addition of N hydrochloric acid.

*Zinc acetate.*—This solution was prepared as 8·1 g. zinc acetate, analytical grade/100 ml. distilled water B.P., giving the same concentration of zinc as above and a pH of 7·0.

*Zinc stearate.*—An emulsion was prepared by mixing 1 g. zinc stearate with 5 ml. acetone and then this suspension was incorporated in 15 ml. of sterile arachis oil.

*Experimental surgical procedures*

The fowl were prepared for inoculation by general anaesthesia with intravenous 2·5 per cent sodium amytal or half-strength veterinary nembutal solution. The apparatus used consisted of a 10 ml. syringe clamped to a stand and connected by fine polythene tubing with the severed stem of a No. 14 or 16 hypodermic needle. This needle introduced into the pectoral vein was strapped in position and the the amount of barbiturate necessary was thus readily controlled.

The testes were exposed by an intercostal approach through the lowest interspace and separation of the ribs by a mastoid retractor. The internal intercostal muscles and then the air sac were incised and 0·20 ml. of the inoculum was injected into the testis by a fine 9 cm. ophthalmic needle. At operation the fowl were numbered serially, with prefixes WL or PL.

In order to detect tumours early, attempts were made by radiography and surgical exploration to diagnose tumour growth before the death of the bird. In the zinc chloride series of 1960, WL 1–40 had lateral radiographs of the abdominal and thoracic cavities.

*Zinc chloride inoculations.*—Total number of fowl inoculated was 159. 111 White Leghorn cockerels aged 5 to 9 months had bilateral intra-testicular inoculation in January and February 1960 and 1961 in Buckinghamshire, England. These birds were kept on open range on mixed corn and mash diet with night arks or house shelter for the hours of darkness. No artificial lighting was used. Forty-eight White Leghorn cockerels had the same type of inoculation in March 1960 and were kept partly on open range but mainly in a well lit wooden poultry house.

*Zinc acetate inoculations.*—Thirty-six White Leghorn cockerels had bilateral intra-testicular inoculation in March 1961. Twenty of these were kept on open range, but due to shortage of space the remaining 16 were kept in cages in a well lit poultry house.

*Zinc stearate inoculations.*—Fourteen White Leghorn cockerels had unilateral intra-testicular inoculation in January and February 1960. Eight received inoculation into the left testis and 6 into the right.

*Transplantation.*—Under aseptic conditions fresh tumour tissue was minced in Hank's balanced salt solution with scalpels in a petri dish into fragments of about 1 mm.$^3$, which were then introduced into the peritoneal cavities and pectoral muscles of young White Leghorn cockerels of the same flock.

### RESULTS

Apart from deaths in the post-operative period the fowl were killed at intervals ranging from 3 weeks to 11 months. The timing of this was partly dictated by the desirability of obtaining the maximum number of tumours for chromosome studies and transplantation.

TABLE I.—*Data Relating to Experimental Avian Teratomas Recorded in the Work of Previous Authors*

| Inoculum | Number of fowl and laterality of testis inoculated: No. | Number of fowl and laterality of testis inoculated: Side | Number, laterality and reference No. of teratomas: Teratomas | Number, laterality and reference No. of teratomas: Side | Number, laterality and reference No. of teratomas: Ref. No. | Size or weight of teratoma | Date of inoculation of tumour bearer | Inoculation/discovery interval (weeks) |
|---|---|---|---|---|---|---|---|---|
| *Michalowsky*, 1926 | | | | | | | | |
| 5% $ZnCl_2$ | ? | ? | 2 | Right | — | 200 g. | Spring, 1925 | 19 |
| | | | | Left | — | 360 g. | Spring, 1925 | 19 |
| *Michalowsky*, 1928 | | | | | | | | |
| 5% $ZnCl_2$ | 130* | Both | 0 | — | — | — | — | — |
| 5% $ZnCl_2$ | 40 | Both | 7† | Right | 158 | 7 cm. long | 1.3.26 | 11 |
| | | | | Left | | 4 cm. long, 20 g. | 1.3.26 | 11 |
| | | | | Right | 171 | Large | 15.3.26 | 11 |
| | | | | Left | | Small nodule within testis | 15.3.26 | 11 |
| *Michalowsky*, 1929 | | | | | | | | |
| 5% $ZnCl_2$ | 12 | Both | 1 | Right | — | 82 g. | ? | 6 |
| *Bagg*, 1936 | | | | | | | | |
| 5% $ZnCl_2$ | 20 | Left | 2 | Left | 256 | 14×9×7·5 cm. | 16.2.33 | 13 |
| | | | | Left | 271 | 10×9×5 cm. | 13.2.33 | 13 |
| 5% $ZnCl_2$+A.P.‡ | 26 | ? | 2 | Left | 485 | 13·5×10·5×6·5 cm. | 7.3.34 | 13 |
| | | | | Right | 296A | 40×95×80 mm. | 11.6.34 | 10 |
| *Bagg*, 1937 | | | | | | | | |
| Autolysate of zinc injected testis+A.P. in 5‡ | 13 | Both | 2 | Left | 443F | 10 mm. diameter | 18.3.— | 6 |
| | | | | Left | 455F | 250 g. | 9.5.— | 7 |
| *Anissimowa*, 1939 | | | | | | | | |
| 5% $ZnCl_2$ | 19 | Both | 2 | Left | — | 204 g. | —.3.37 | 13 |
| | | | | Left | — | 230 g. | —.3.37 | 15 |
| *Falin and Gromzewa*, 1939 | | | | | | | | |
| 10% $ZnSO_4$ | 22 | Both | 3 | Right | 5797 | 66·15 g. | 17.3.38 | 12 |
| | | | | Left | 1823 | 238 g. | 28.3.38 | 8 |
| | | | | Right | 492 | 91·5 g. | 28.3.38 | 10 |
| 10% $ZnSO_4$ | 7§ | Both | 0 | — | — | — | — | — |
| *Falin and Anissimowa*, 1940 | | | | | | | | |
| 5% $CuSO_4$ | 17 | Both | 1 | Left | — | 79 g. | 13.3.39 | 10 |
| 5% $CuSO_4$ | 21§ | Both | 0 | — | — | — | — | — |
| *Falin and Gromzewa*, 1940*a* | | | | | | | | |
| 10% $ZnSO_4$ | 29 | Both | 1 | Left | — | 6·25 g. | 28.3.38 | 22 |
| 10% $ZnSO_4$ | 43 | Both | 1 | Left | — | 0·4 g. | 19.3.39 | 11 |
| *Falin and Gromzewa*, 1940*b* | | | | | | | | |
| 10% $ZnNO_3$ | 30 | Both | 5 | Both | — | 0·5 g. and 1·0 g. | 17.3.39 | 6 |
| | | | | Right | — | 8·3 g. | 3.4.39 | 9 |
| | | | | Left | — | 7·5 g. | 3.4.39 | 12 |
| | | | | Left | — | 4·8 g. | 17.3.39 | 12 |

TABLE I.—*continued.*

| Inoculum | Number of fowl and laterality of testis inoculated | | Number, laterality and reference No. of teratomas | | | Size or weight of teratoma | Date of inoculation of tumour bearer | Inoculation/discovery interval (weeks) |
|---|---|---|---|---|---|---|---|---|
| | No. | Side | Teratomas | Side | Ref. No. | | | |
| *Falin*, 1940 | | | | | | | | |
| 5% $ZnCl_2$ | ? | ? | 6 | ? | 5349 | 0·6 g. | ? | 4 |
| | | | | ? | 1800 | 1·0 g. | ? | 6 |
| | | | | Right | 4383 | 20 g. | ? | 6 |
| | | | | Right | 4309 | 3·2 g. | ? | 6 |
| | | | | ? | 141 | 162 g. | ? | 21 |
| | | | | ? | 2743 | 202 g. | ? | 21 |
| *Carleton, Friedman and Bomze*, 1953 | | | | | | | | |
| 5% $ZnCl_2$ | ? | ? | ? | ? | — | ? | ? | ? |
| 5% $ZnCl_2$ | 43 | Both | 11 | ? | — | ? | ? | ? |
| *Smith and Powell*, 1957 | | | | | | | | |
| 10% $ZnSO_4$ | 7 | Right | 0 | — | — | — | — | — |
| 5% $ZnCl_2$ | 9 | Right | 0 | — | — | — | — | — |
| 5% $ZnCl_2$ | 8 | Both | 1 | ? | — | ? | —.2.— | ? |

* May–December inoculation.
† Details of 3 were not recorded by Michalowsky.
‡ A.P. = anterior pituitary extract.
§ April inoculation.

### *Zinc Chloride Inoculations*

Satisfactory X-ray examinations involved general anaesthesia and were discontinued after the first 40 examinations, WL 1–40, as surgical exploration took little longer, was more certain in diagnosis, and had negligible mortality. Nevertheless, radiographs did reveal the larger tumours as seen in Fig. 7 and with improved technique might have shown earlier tumours.

Fig. 2 relates the time of discovery of the tumour or death of the bird to the time of inoculation of the zinc chloride solution. In the 111 fowl receiving

FIG. 2.—Diagram relating time of discovery of teratoma to date of inoculation.

bilateral inoculations in the months of January and February, 8 teratomas were induced, a tumour incidence of 7·2 per cent of the birds inoculated and 3·6 per cent of the testes inoculated. But in the 48 fowl inoculated in March, no tumours were induced.

Out of the 318 testes injected, 296 including the 8 tumour-bearing testes showed necrosis of tubules, blood clot or fibrous scarring at post mortem examination. No lesions were seen in 22. This may have been due to the inoculum leaking out immediately after the injection.

The injection of 0·2 ml. of the zinc chloride solution produced a blanching and swelling of part of the organ and in some cases immediate subcapsular haemorrhage. From a study of the early post-operative deaths, about one quarter to one third of the testis showed an opaque whitish appearance within 12 hours of the injection. In all the deaths within the first three post-operative weeks this was complicated by blood clot in the testes and in the adjoining part of the abdominal air sacs. Histologically the changes of necrosis, pyknosis and then gradual loss of nuclear staining were clearly evident from 12 hours onwards. Inside the necrotic zone all cells of the seminiferous tubules, interstitium and blood vessel walls had undergone necrosis, but the outline of these cells and structures remained for several months. PL 21 is a typical example at 7 weeks post-inoculation and Fig. 1 shows the histological appearance. This " freezing " of the histological appearances at the time of inoculation is of interest, as the rest of the testis continued to undergo the normal spring and early summer development. At an early stage the necrotic area became surrounded by multinucleated giant cells and a lymphocytic exudate (WL 38, Fig. 3). Tubules adjacent to the

### EXPLANATION OF PLATES

Fig. 1.—PL 21. Section of testis showing centrally the almost unstained necrotic area at site of zinc chloride inoculation. H. and E. ×35.

Fig. 3.—WL 38. Section of testis showing, at the bottom, multinucleated giant cell and lymphocytic reaction around necrotic tubules. The tubules at the top show a few cells with giant nuclei. H. and E. ×100.

Fig. 4.—PL 27. Section of left testis showing at top right an encysted keratinized area of air sac epithelium, then a homogeneous pear-shaped area of necrotic tubules, and below this the teratoid tumour. H. and E. ×4·2.

Fig. 5.—WL 85. Section of left testis showing teratoid tumour at the top with zones of lymphocytes separating it from the normal seminiferous tubules below. H. and E. ×62.

Fig. 7.—WL 37. Lateral radiograph showing rounded opacity due to teratoma testis anterior to dorsal spine. ×4/7.

Fig. 8.—WL 43. Both testes bisected and opened out. The halves of right testis with teratoma are central, and the left testis showing central clot is on the outside. The relatively unaffected poles of right testis are clearly seen.

Fig. 9.—PL 10. Histological appearance of seminiferous tubules at 5 weeks after winter solstice. Note large numbers of spermatogonia and absence of mature forms of gametes. H. and E. ×202.

Fig. 10.—Testis of normal White Leghorn cockerel at 11 weeks showing advanced spermiogenesis. H. and E. ×182.

Fig. 11.—WL 43. Section of left testis showing intestinal type of epithelium forming cysts and tubules in proliferating mesenchyme. Remains of blood clot are seen at the bottom, and seminiferous tubules at the top. Striped muscle fibres separate the growth from the clot. H. and E. ×56.

Fig. 12.—WL 74. Section of right-sided teratoma showing, below, tumour consisting of medullary type epithelium and cerebral tissue, nerve cells and astrocytes. H. and E. ×44.

Fig. 13.—WL 74. High power view of section in Fig. 12 showing nerve cells and smaller astrocytes. H. and E. ×192.

1

3

4

5

7

8

9

10

11

12

13

necrotic zone showed increased numbers of multinucleated spermatocyte complexes and occasional giant cells with large convoluted nuclei in the lumina, but despite the examination of large numbers of sections no exclusively intra-tubular neoplasia was seen. Blood clot and coagulated seminiferous tubules were both surrounded by multinucleated giant cells, lymphocytes and numerous histiocytes containing blood pigments of golden yellow, greenish yellow and dark brown

FIG. 6.—Diagram illustrating the relationship between times of inoculation, winter solstice and resultant production of teratomas.

colour. A proportion of this pigment, mainly the dark brown, gave a positive reaction for iron with Perls and Turnbull's method. In several cases, and in one conspicuously (WL 38), heavy deposits of iron could be demonstrated in the exfoliated cells lying in the lumina of adjacent tubules. This was almost certainly due to haemorrhage into the tubules. A proportion of the birds had blood staining of the droppings for several days post-operatively. Later fibrous scarring leading eventually to an hour-glass appearance predominated at the site of inoculation, although the solidified blood clot remained in several cases for over 8 months. Fig. 6 illustrates the relationship between time of inoculation and winter solstice (December 21) and the numbers developing teratomas in each inoculation group is shown.

The 8 tumours produced were all teratomas and consisted of varying proportions and arrangements of embryonic and adult tissues. The complexity of their structure cannot be described here, and a full topographical study has not yet been undertaken, but they resembled the previous experimental testicular teratomas induced in cockerels by Michalowsky (1926) and later authors. Table II shows the main characteristics. Six out of the 8 teratomas arose in the right testis. The other 2 were very small growths and arose in the left testis. The largest tumour, in WL 37, replaced the greater part of the testis, but the others

TABLE II.—*Data Relating to Experimental Teratomas Induced by Zinc Chloride (Present Series)*

| Bird | Testis showing tumour | Date of inocula-tion | Winter Solstice/ inoculation interval (weeks) | Date tumour dis-covered | Inocula-tion/ discovery interval (weeks) | Tumour volume (c.c.) | State of tubules* Tumour-bearing testis | State of tubules* Other testis | Adeno-hypo-physis† |
|---|---|---|---|---|---|---|---|---|---|
| WL 37 | Right | 13.1.60 | 4 | 17.5.60 | 18 | 220 | A.S. | A.S. | β cells + |
| WL 63 | Right | 5.2.60 | 7 | 7.6.60 | 17 | 110 | Early summer steatogenesis | | β cells + |
| WL 70 | Right | 5.2.60 | 7 | 13.4.60 | 10 | 100 | D.S. | A.S. | β cells + |
| WL 74 | Right | 8.2.60 | 7 | 27.4.60 | 11 | 70 | A.S. | A.S. | — |
| WL 43 | Right (+ doubtful microscopic focus in left) | 20.1.60 | 5 | 3.4.60 | 10 | 60 | A.S. | A.S. | — |
| PL 26 | Right | 30.1.61 | 6 | 16.3.61 | 6 | 140 | A.S. | A.S. | β cells + |
| PL 27 | Left | 30.1.61 | 6 | 16.3.61 | 6 | 0·05 | A.S. | A.S. | β cells + |
| WL 85 | Left | 25.2.60 | 10 | 28.7.60 | 22 | 0·10 | A.S. | A.S. | — |

* A.S. = active spermiogenesis. D.S. = depressed spermiogenesis.
† β cells + = cells, showing granules stained purple by P.A.S. technique and not stained by Gömöri's aldehyde fuchsin stain, present in at least normal numbers.

appeared to arise from the central part of the organ, close to the necrotic area or blood clot (WL 43, Fig. 8). In one case, WL 70, the teratoma grew out in both directions in dumb-bell form. The relationship of one of the minute teratomas to the solid area of coagulated tubules is well illustrated in PL 27 (Fig. 4) where the growth is of the nature of embryonal carcinoma with early teratoid differentiation. Fig. 4 also shows a cystic area where the air sac epithelium has undergone squamous change with keratinization.

The other small growth, in WL 85, was discovered only after section of the testis when the somewhat opaque and solid appearance of the upper pole was noticed. This also was of the nature of embryonal carcinoma with early formation of horn pearls and primitive endodermal tubules (Fig. 5). Although found 22 weeks after inoculation it showed a high mitotic rate and infiltration of testicular capsule.

With the exception of one of the small tumours, the other teratomas arose from inoculations made from 4 to 7 weeks after the winter solstice. The testis of the domestic fowl at this stage shows active division of spermatogonia and the resultant spermatogonial layer with primary spermatocytes is 3 to 4 cells thick (Fig. 9). Later at 10 to 16 weeks spermiogenesis is in full flood and spermatogonial divisions are not conspicuous (Fig. 10).

The testicular capsule is normally covered by the ciliated columnar epithelium of the air sac. In two cases this showed cystic invagination at the injection site with squamous transformation and metaplasia. In the left testis of one of these, WL 43, which had a right-sided teratoma (Fig. 8), this type of lesion progressed a stage further and here horn cysts continued into a cyst lined by copiously mucin secreting non-ciliated epithelium of intestinal type. This cyst showed buds extending out into a mesenchyme heavily infiltrated by lymphoid cells and containing a few bands of smooth muscle (Fig. 11).

### *Zinc Acetate Inoculations*

Thirty-six White Leghorn cockerels received bilateral intra-testicular injections in March 1961 (Fig. 6). Rather smaller areas of necrosis resulted and haemorrhage was less conspicuous. There were no immediate post-operative deaths and to allow the maximum number of neoplasms to develop most of the birds were allowed to survive 6 to 11 months. All these birds showed fibrous scars, the earlier ones being infiltrated with lymphocytes, and 17 also showed blood pigments in histiocytes. One showed in the scar several conspicuous lymphoid foci with centres like germinal centres and isolated foci of similar reticulum-like cells. These were similar to those described by Smith and Powell (1957). Two showed horn cysts at the injection sites, and two others showed Sertoli cell nodules, but no frank tumours were found.

### *Zinc Stearate Inoculations*

The injection of this emulsion into the testes of 17 cockerels in January and February 1960 produced small cystic lesions surrounded by giant cells and lymphocytes. There was minimal testicular scarring when birds were killed 3 to 6 months later. No tumours were found.

### *Pituitary Gland*

The pituitary gland was removed post mortem from 26 fowl in the zinc chloride series, including 5 out of the 8 tumour bearers, and from 4 in the zinc acetate series. It was fixed in 10 per cent neutral formalin and serial paraffin sections stained at intervals with haematoxylin and eosin, periodic acid-Schiff (P.A.S.) method and Gömöri's aldehyde fuchsin. The normal histology of the avian pituitary gland is rather different from the mammalian one. Recent reports include those of Mikami (1958), Herlant *et al.* (1960) and Tixier-Vidal (1962). There is still considerable lack of agreement about terminology, but Herlant and his associates recognise 6 granular types including the P.A.S. positive, and aldehyde fuchsin negative $\beta$ cells, which they consider to be secretors of follicle stimulating hormone. The variation of the cytological picture according to the time of year is well recognised, and from the author's preliminary study of the pituitary gland in normal fowl there also appears to be variation due to alteration of lighting conditions.

The pars anterior of the 5 tumour bearers whose pituitary glands were available showed $\beta$ cells in apparently normal numbers (Table II). All these birds showed active spermiogenesis, although one had early summer steatogenesis. Comparison with non-tumour bearers showing similar testicular conditions revealed no histological differences with the staining techniques employed. A case of spontaneous testicular teratoma in a fowl at present being studied by the author has shown no P.A.S. positive granulation in an enlarged pituitary gland.

*Transplantation*

Attempts were made to transplant teratomas by implanting fragments of teratomas from 5 birds (WL 37, WL 70, WL 74, PL 26 and PL 27) each into a pair of cockerels. From WL 37 and PL 26 transplantation was made into the peritoneal cavity only. In the others, fragments were also implanted into pectoral muscle. Although 10 and 12 weeks later nodules of cartilage were recovered in the peritoneum of two recipients of teratomas from WL 37 and PL 26 there was no evidence of growth. No trace of the transplants from the other teratomas was found.

### DISCUSSION

The literature has consistently stressed the necessity of injecting the zinc salts in the spring and all authors have specified the months January to March. Bagg (1936) however was able to induce a teratoma out of season by using mammalian anterior pituitary extract. In these early spring months spermatogenetic activity of fowl as evidenced by gonadal size and histological examination has been found by the author to vary considerably, especially in birds which have recently reached sexual maturity. Lighting conditions in the preceding months as well as in the period under study, temperature and diet all have an influence. About 20 per cent of the testes inoculated in January were less than 1·0 cm. in transverse diameter. During March, i.e. 10 to 14 weeks after the winter solstice, all testes inoculated with zinc chloride and acetate were large, about 2·0 cm. in transverse diameter, and examination of 3 early post-operative deaths not included in Fig. 6 showed established spermiogenesis throughout the tubules. The 8 teratomas in the present series were induced by injections given between 4 and 10 weeks after the winter solstice and 7 of these between 4 and 7 weeks when the testis showed maximal spermatogonial division (Fig. 9) with only short segments of tubules exhibiting spermiogenesis. Although previous workers have produced teratomas from March inoculations, the present failure to induce them in 48 cockerels inoculated with zinc chloride in March is not statistically significant in view of the unexpectedly low percentage of teratomas induced in January and February. Higher incidence of teratomas, up to 25 per cent in certain groups, was reported by the American workers and Falin and associates in Russia. This low tumour incidence in the present series of zinc chloride inoculation also reduces the significance of the failure of zinc acetate to induce teratomas in 36 White Leghorn cockerels. Although no teratomas were induced, a much larger series would be required for the exclusion of zinc acetate as a carcinogen.

According to Willis (1960) human testicular teratomas occur more frequently on the right side. The present series (Table II) contains 6 right-sided tumours varying from 60 c.c. to 220 c.c. in volume and only 2 left-sided teratomas, both minute in size. These dwarf tumours, WL 85 and PL 27, showed almost entirely the monocellular extra-tubular growth of embryonal carcinoma with foci of early somatic differentiation (Fig. 5), while the larger tumours consisted mainly of differentiated embryonic and adult tissues (Fig. 12 and 13). These dwarf left-sided tumours were not early tumours, unless they possessed a long latent period before the initiation of growth, as one was discovered 6 weeks and the other 22 weeks after inoculation. Because of the small numbers involved in the present series the significance of the production of these dwarf tumours in the left testis is difficult to assess. Table I shows that various units of measurement have been used by the previous authors. The weight of the tumour in most cases includes the weight of the testis and this is more significant in the case of the smaller tumours. They do, however, fall into two distinct groups : (*a*) those clearly above the normal testicular size and weight for mid-summer, when nearly all the tumours were harvested, i.e. 20 g. and above, 18 c.c. and above, and over 3·5 cm. in length ; (*b*) those below these measurements, dwarf teratomas. These are tabulated in Table III. The figures show that 17·6 per cent of right-sided and

TABLE III.—*Laterality of Large and Dwarf Experimental Teratomas Recorded to Date, Including the Present Series*

| | Large teratomas (above normal testicular size or weight) | Dwarf teratomas (below normal testicular size or weight) | Total number of measured teratomas of known laterality* |
|---|---|---|---|
| Right testis | 14 | 3 | 17 |
| Left testis | 10 | 9 | 19 |
| Total | 24 | 12 | 36 |
| Interval between inoculation and discovery of tumour (mean and S.D.) (weeks) | $11 \cdot 6 \pm 2 \cdot 7$ | $10 \cdot 75 \pm 5 \cdot 6$ | — |

* There was no record of the size and laterality of 19 other recorded experimental teratomas.

47·4 per cent of left-sided teratomas are in this dwarf category (group *b*), a difference of 29·8 per cent. The standard error of the difference between these proportions is 14·6. The actual difference is just over twice this and thus the closer association of dwarf teratomas with the left side is not likely to be fortuitous. It will be noted from Table III that left-sided teratomas were not harvested at a shorter period after inoculation than right-sided tumours. The significance of any such behavioural difference of tumour growth or induction between left and right gonad is not clear. The left testis in fowl is usually larger than the right (van Tienhoven, 1961) and the writer has noted this in the White Leghorn fowl used in these experiments and in the chick embryo when the left testis is longer and narrower. Macartney (1942) has commented on the larger number of mitotic figures in the right testis. It should also be remembered that the female in many species of bird is well known for the asymmetrical development of its gonads.

No observations were made on the rate of growth of these tumours, as they were found at post mortem examination or when detected during life the animals were killed within 24 hours. The interval between inoculation and initiation of neoplasia may have varied considerably from case to case. The right-sided teratoma of PL 26, although containing areas of embryonal carcinoma, consisted mainly of adult and embryonic structures at 6 weeks after inoculation and was 140 c.c. in volume, while the left-sided teratoma of PL 27, discovered at the same interval after inoculation, was almost entirely embryonic carcinoma and 0·05 c.c. in volume. Again the embryonic carcinoma of WL 85, showing histological evidence of rapid growth and infiltration, was 0·10 c.c. in volume at 22 weeks after inoculation. It can be seen from Table II that there is no correlation between size of tumour and the period elapsing since inoculation. In neither group in the present series could exclusively intra-tubular neoplasia be found and although adjacent tubules here and in other testes of the zinc chloride series showed spermatogonial proliferation with giant cell formation, none of these intra-tubular cells could be identified cytologically with the known tumour cells of embryonal carcinoma. Derivation of these avian teratomas from germ cells may be an acceptable thesis, but does not demand that these teratomas commence as intra-tubular growths. They may well be derived from extra-tubular germ cells, sited developmentally or as a result of damage to the tubular basement membrane. It must be remembered that primordial germ cells migrate to the developing gonads from the region of the yolk sac (Witschi, 1948) and, in the chick, Simon (1960) has shown that they travel a considerable distance in the blood stream. Smith and Powell (1957) have questioned the identity of large reticulum-like cells in the interstitial tissues both in normal controls and in zinc inoculated testes and suggested that these may be primordial germ cells. Also the cysts at the injection site found in two cases in the zinc chloride and in two cases in the zinc acetate series raise again the question of the inducing capacity of the ectodermally derived respiratory epithelium of the air sacs. Attempts to induce avian teratomas by injection of metallic salts into the abdominal organs may prove of value. Gaillard (1962), who supported an extra-tubular origin of teratomas, described large embryonal type nuclei in the interstitial tissues of human testis, tumourous and non-tumourous. He believed that germ cells migrated out of the tubules and exchanged information with these embryonal nuclei, which then became neoplastic and formed the basis of teratomas. In his studies of the spontaneous testicular teratomas of strain 129 mice he claimed that he observed these extra-tubular embryonic nuclei in infant mice bearing tumours. Stevens (1962) who with Little (Stevens and Little, 1954) originally described spontaneous testicular teratomas in strain 129 mice could not agree with Gaillard's interpretation and illustrated foci of proliferating cells within seminiferous tubules. He believed that these were early stages of the teratocarcinoma.

With the experimental teratomas this intra-tubular origin remains unproved.

One of the theories of induction of these teratomas advanced by Falin was that the zinc salts liberated inductors from the dead cells in the necrotic area and that these acted on the totipotent germ cells. Bagg's experiments are of interest in this connection in that he produced two teratomas with the autolysate from testes inoculated with zinc chloride solution 4 or 5 days previously (Bagg, 1937). This procedure is open to the objection that the zinc is still present, and we know from our studies with $^{65}ZnCl_2$ (Guthrie, 1962b, and unpublished data) that an appreciable quantity is still present in the inoculated area at this period. This zinc is almost certainly strongly chelated by nucleic acids, proteins, peptides and amino acids. It is very difficult to remove without interfering with these chelating groups.

Apart from the possible role of the zinc, iron cannot be excluded as a carcinogen here. It is present in considerable amounts both in tubules and in the interstitium near the site of necrosis and haemorrhage.

Champy and Lavedan (1939) considered that Michalowsky's teratomas were the sequel of partial castration and resultant hypophyseal stimulation. Gonadotrophin estimation in tumour bearers might provide this evidence, but histological examination of the pituitary glands of the tumour bearers in the present series has not provided any evidence in support of this so far. Further examination including a wider range of controls will be necessary and the effects of hypophysectomy will need to be ascertained. Interstitial cell tumours may be produced in rodents by testicular damage, for example by implanting testis into the spleen (Jones, 1955) and other procedures associated with degeneration of the seminiferous tubules (Guthrie, 1956). No interstitial cell tumours arose in the present series, although small nodules of tubules lined by Sertoli cells were present. Incidentally interstitial cell tumours have not to the author's knowledge been described in domestic fowl, although they are known in other species of the class Aves.

SUMMARY

Eight teratomas developed in the testes of 111 White Leghorn cockerels which had received bilateral intra-testicular inoculations of 5 per cent zinc chloride solution in the months of January and February. These inoculations produced zones of haemorrhagic necrosis in the testis, eventually leading to scars, pigmented with iron and other pigments from broken down haemoglobin. Teratomas arose close to the area of injury, but although intra-tubular proliferation of spermatogonia and spermatocytes was seen, no exclusive intra-tubular neoplasia was detected. Two cases showed horn cysts and proliferation of mucin secreting non-ciliated epithelium in proliferating mesenchyme close to the blood clot at the site of injection. The possible role of iron as a carcinogen is mentioned. Six of the tumours were several times the size of the normal testis and had arisen from the right testis. Two of the tumours were minute and did not increase testicular size. They arose from the left testis. Taken together with the published records, analysis shows that there is a significant association of these small teratomas with the left testis.

Forty-eight White Leghorn cockerels similarly inoculated with zinc chloride in March produced no tumours.

Thirty-six White Leghorn cockerels inoculated intra-testicularly with 8·1 per cent zinc acetate solution of pH 7·0 in March showed less severe scarring than those inoculated with zinc chloride and no tumours.

A group of 14 cockerels of the same breed had intra-testicular inoculations of zinc stearate in January and February. This produced small cysts, no appreciable necrosis or scarring, and no tumours.

The pituitary glands examined showed no significant differences between tumour bearers and non-tumour bearers. They did not show the appearances associated with recent castration.

My thanks are due to Professor A. Haddow, F.R.S., Director, Chester Beatty Research Institute, London, for facilities at Pollards Wood Research Station. To Miss W. M. Gallagher I am indebted for the translation of the articles in German. The work has been generously financed by the British Empire Cancer Campaign.

### REFERENCES

Anissimowa, W. W.—(1939) *Amer. J. Cancer*, **36**, 299.

Bagg, H. J.—(1936) *Ibid.*, **26**, 69.—(1937) *Science*, **85** : Supplement No. 4, p. 92.

Bresler, V. M.—(1959) *Probl. Oncol.*, **5**, 24.—(1962) 'VIIIth International Cancer Congress Abstracts (Moscow, 1962)'. Moscow (Medgiz), p. 202.

Campbell, J. G.—(1951) *Brit. J. Cancer*, **5**, 69.

CARLETON, R. L., FRIEDMAN, N. B. AND BOMZE, E. J.—(1953) *Cancer*, **6**, 464.
CHAMPY, C. AND LAVEDAN, J. P.—(1939) *Bull. Ass. franç. Cancer*, **28**, 503.
FALIN, L. I.—(1940) *Amer. J. Cancer*, **38**, 199.—(1941) *Z. mikr-anat. Forsch.*, **49**, 193.
*Idem* AND ANISSIMOWA, W. W.—(1940) *Z. Krebsforsch.*, **50**, 399.
*Idem* AND GROMZEWA, K. E.—(1939) *Amer. J. Cancer*, **36**, 333.—(1940a) *Virchows Arch.*, **306**, 300.—(1940*b*) *Ibid.*, **306**, 578.
GAILLARD, J. A.—(1962) *Arch. Anat. path.*, *Paris*, **10**, 31.
GUTHRIE, J.—(1956) *Brit. J. Cancer*, **10**, 134.—(1962a) *Exp. Cell Res.*, **26**, 304.—(1962b) *Rep. Brit. Emp. Cancer Campgn*, **40**, 289.
HERLANT, M., BENOIT, J., TIXIER-VIDAL, A. AND ASSENMACHER, I.—(1960) *C.R. Acad. Sci.*, *Paris*, **250**, 2936.
JONES, A.—(1955) *Brit. J. Cancer*, **9**, 640.
MACARTNEY, E. L.—(1942) *Poult. Sci.*, **21**, 130.
MASHAR, U.—(1932) *Virchows Arch.*, **285**, 155.
MICHALOWSKY, I.—(1926) *Zbl. allg. Path. path. Anat.*, **38**, 585.—(1928) *Virchows Arch.*, **267**, 27.—(1929) *Ibid.*, **274**, 319.
MIKAMI, S. I.—(1958) *J. Fac. Agric.*, *Iwate Univ.*, **3**, 473.
SIMON, D.—(1960) *Arch. Anat. micr. Morph. exp.*, **49**, 93.
SMITH, A. G. AND POWELL, L.—(1957) *Amer. J. Path.*, **33**, 653.
STEVENS, L. C.—(1962) *Ann. Biol.*, **1**, 586.
*Idem* AND LITTLE, C. C.—(1954) *Proc. nat. Acad. Sci.*, *Wash.*, **40**, 1080.
VAN TIENHOVEN, A. M.—(1961) in ' Sex and Internal Secretions ', 3rd edition. Edited by W. C. Young. London (Baillière, Tindall and Cox), Vol. 2, p. 1089.
TIXIER-VIDAL, A.—(1962) *Biol. méd.*, *Paris*, **51**, 183.
WILLIS, R. A.—(1960) ' Pathology of Tumours ', 3rd edition. London (Butterworth), p. 952.
WITSCHI, E.—(1948) *Contr. Embryol. Carneg. Instn*, **32**, 67.


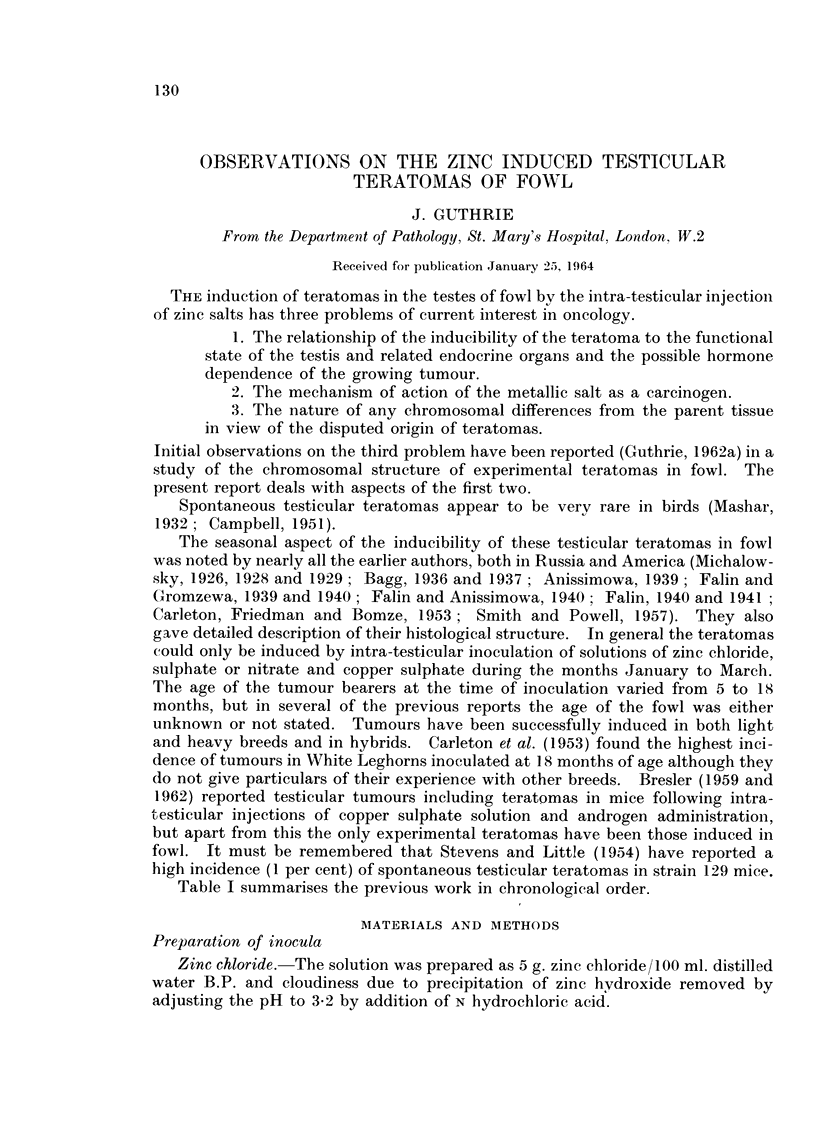

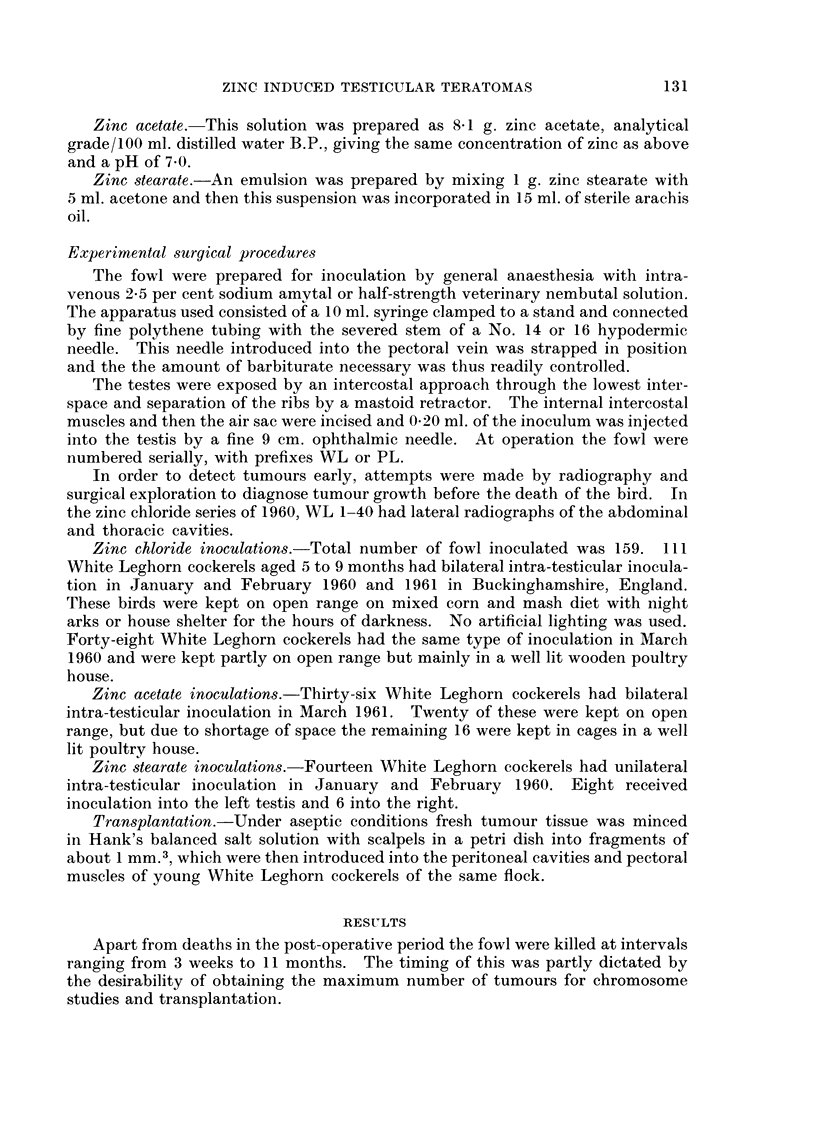

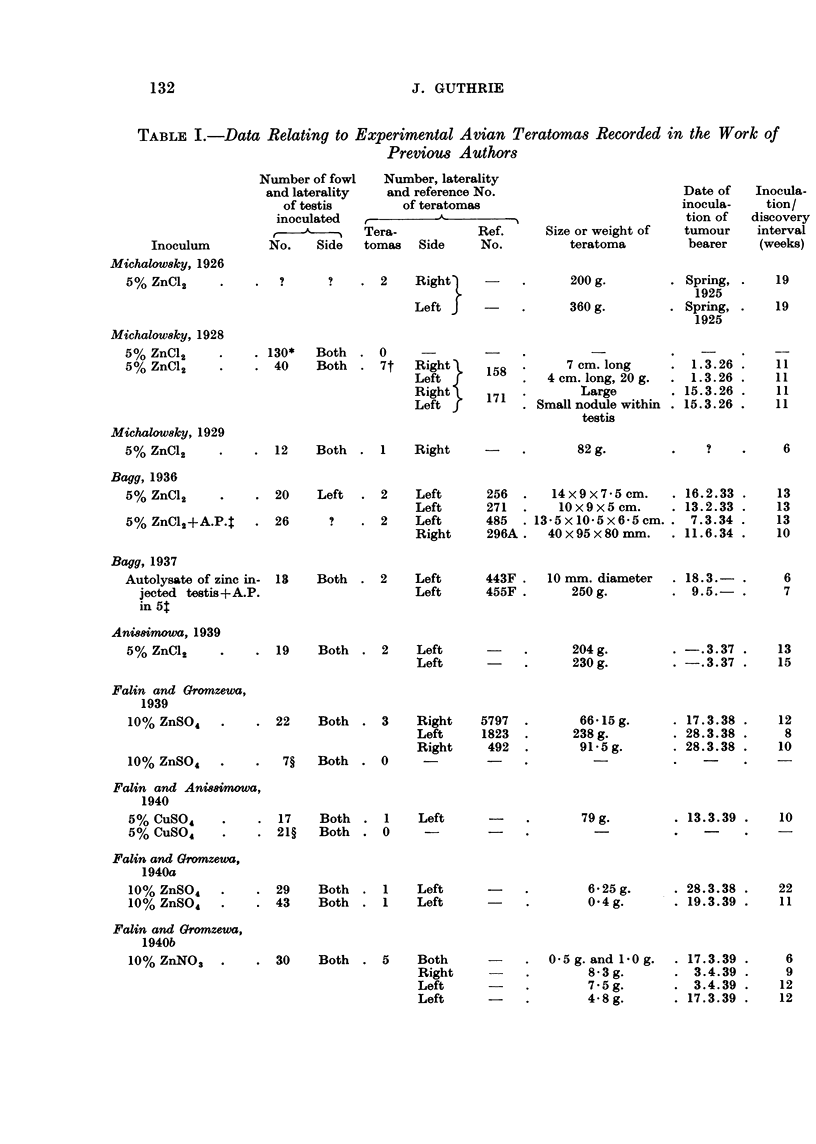

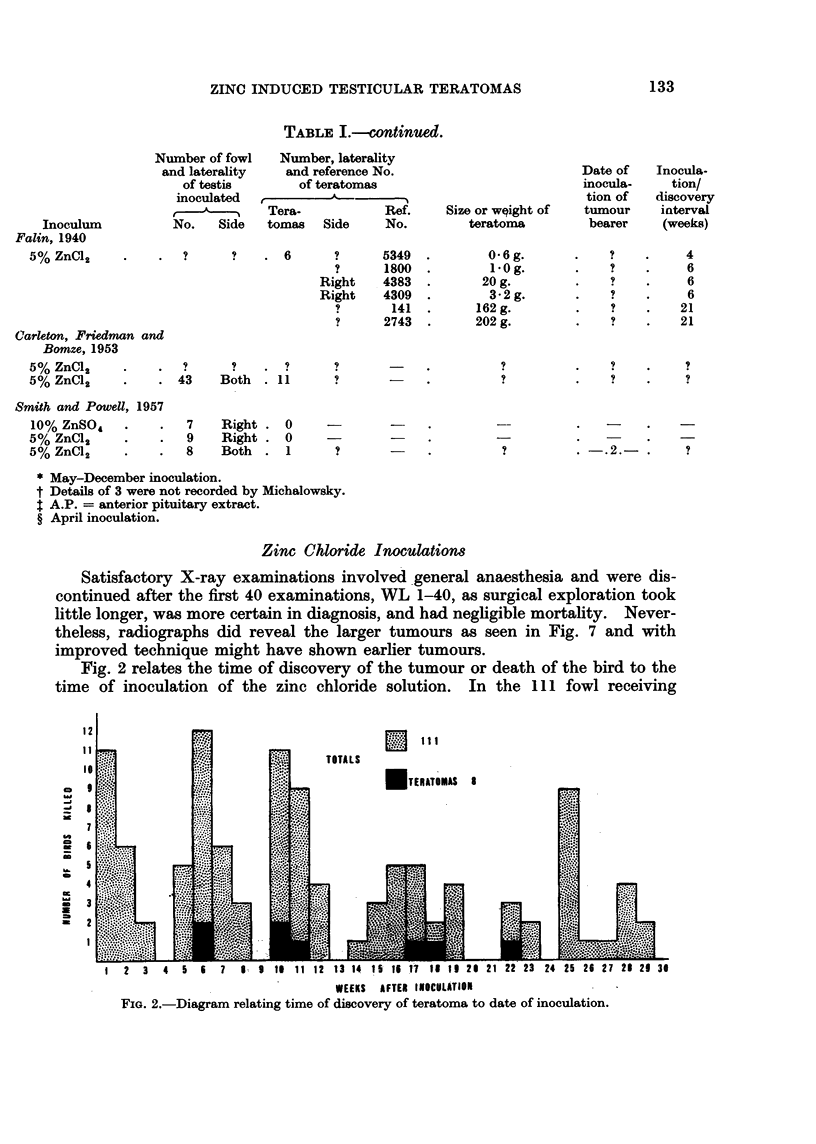

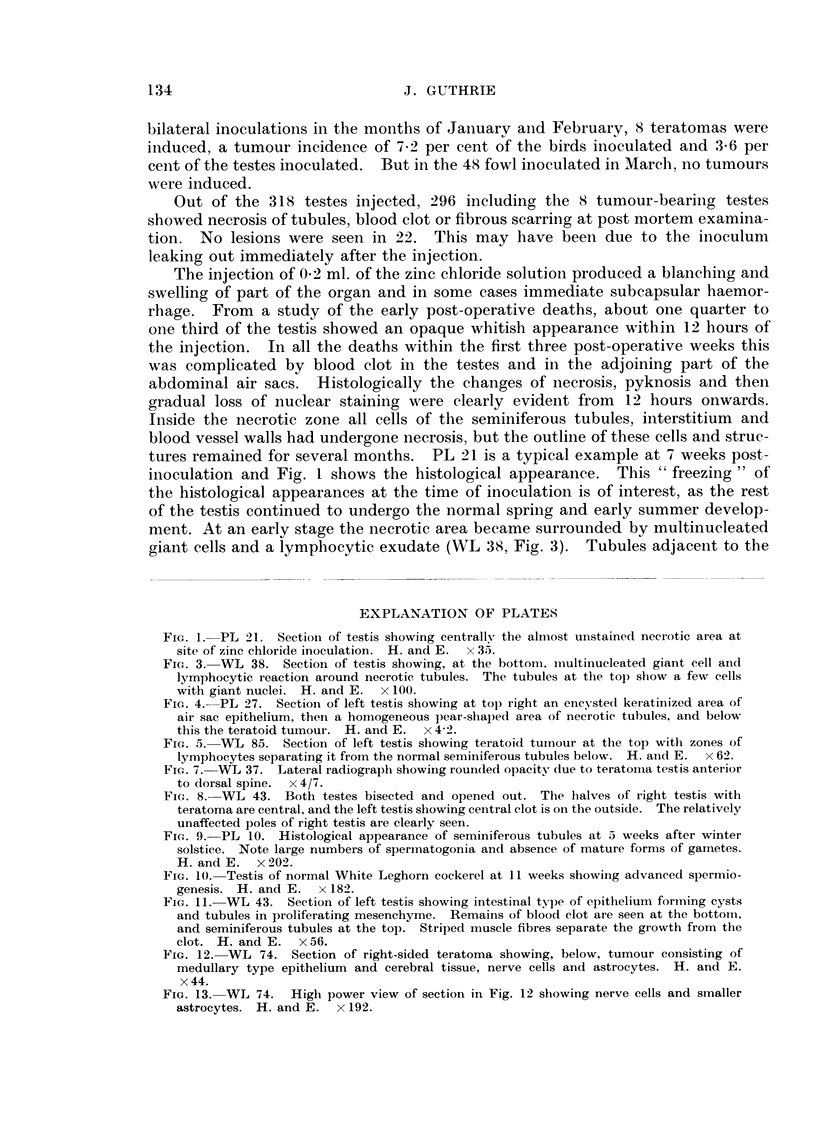

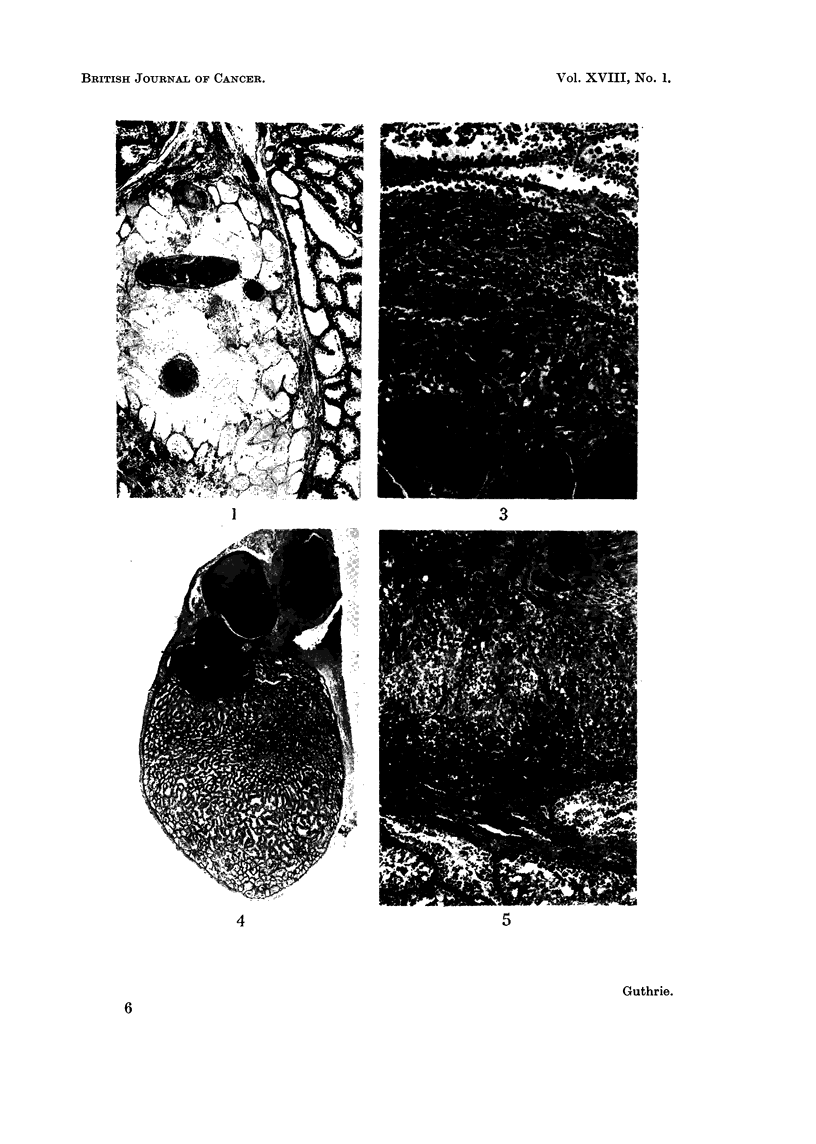

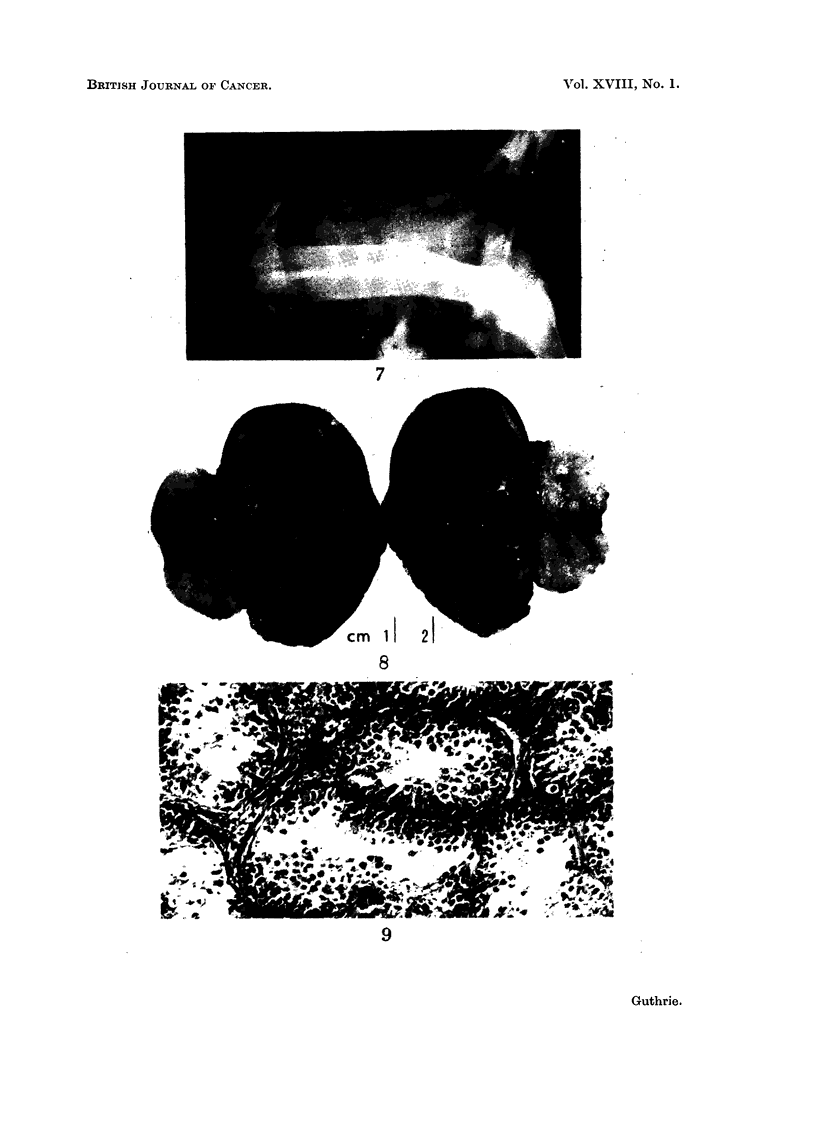

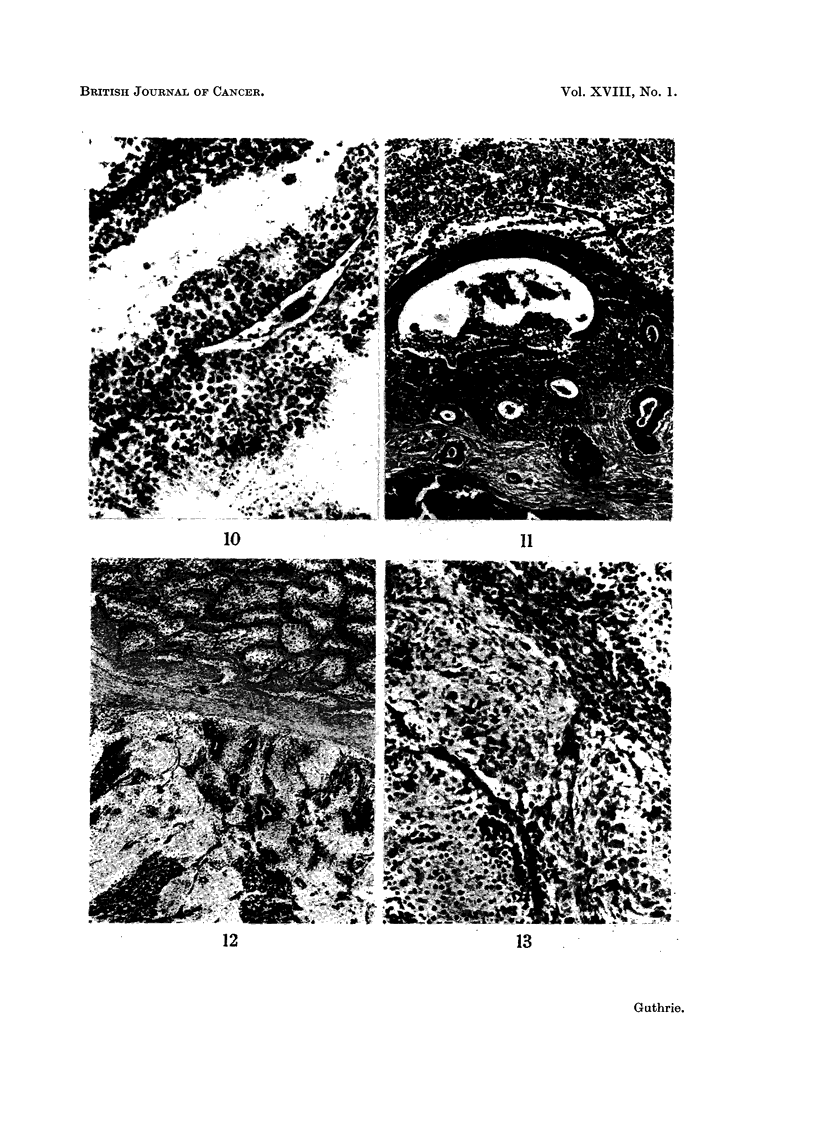

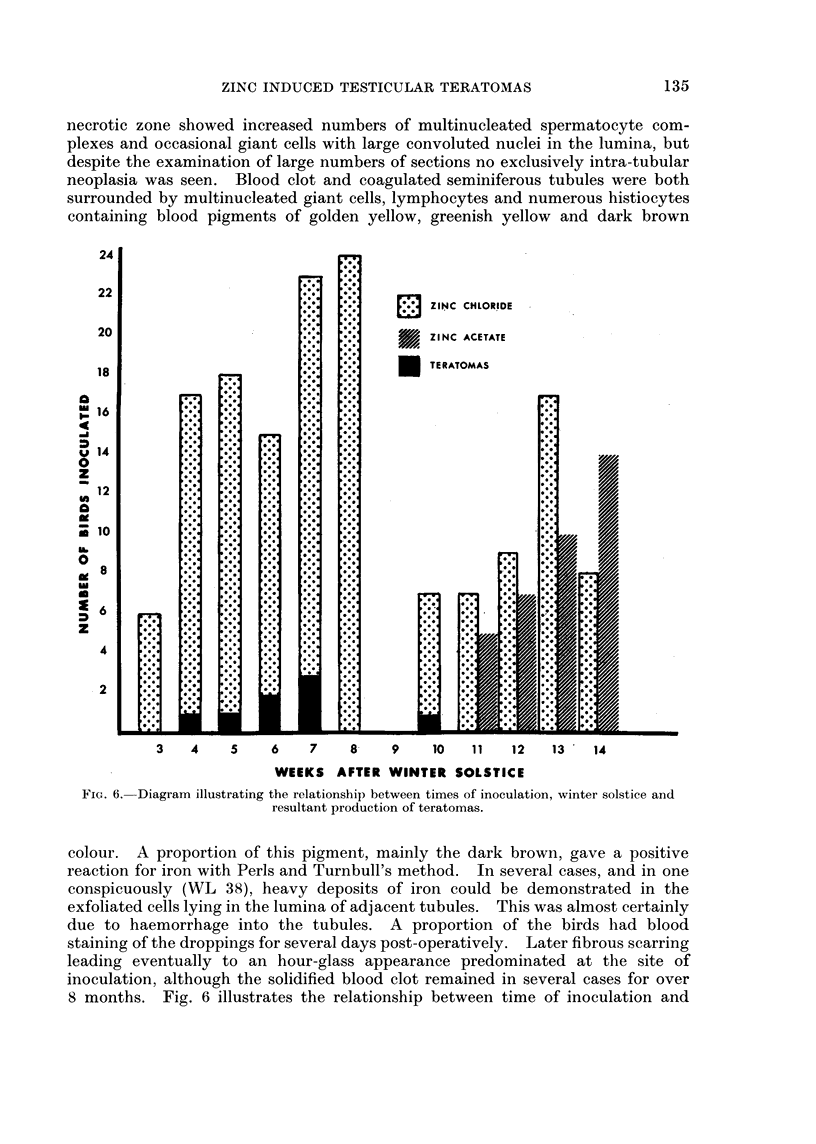

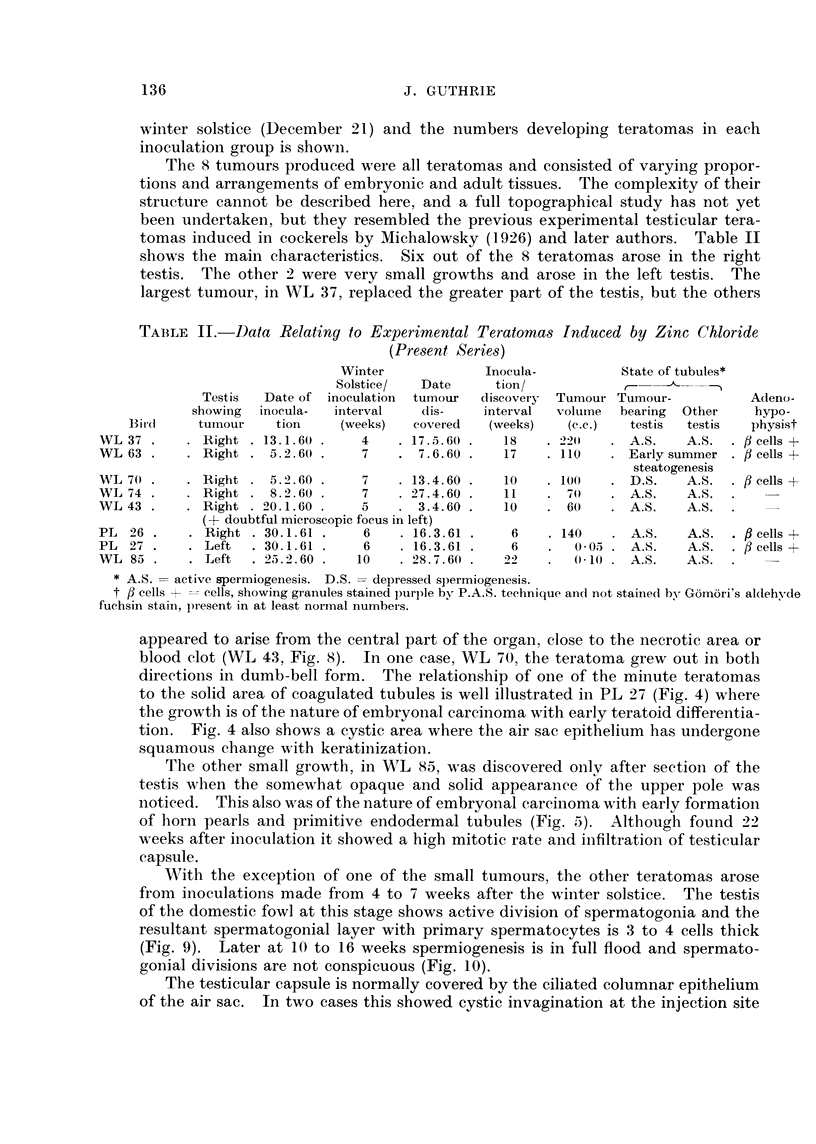

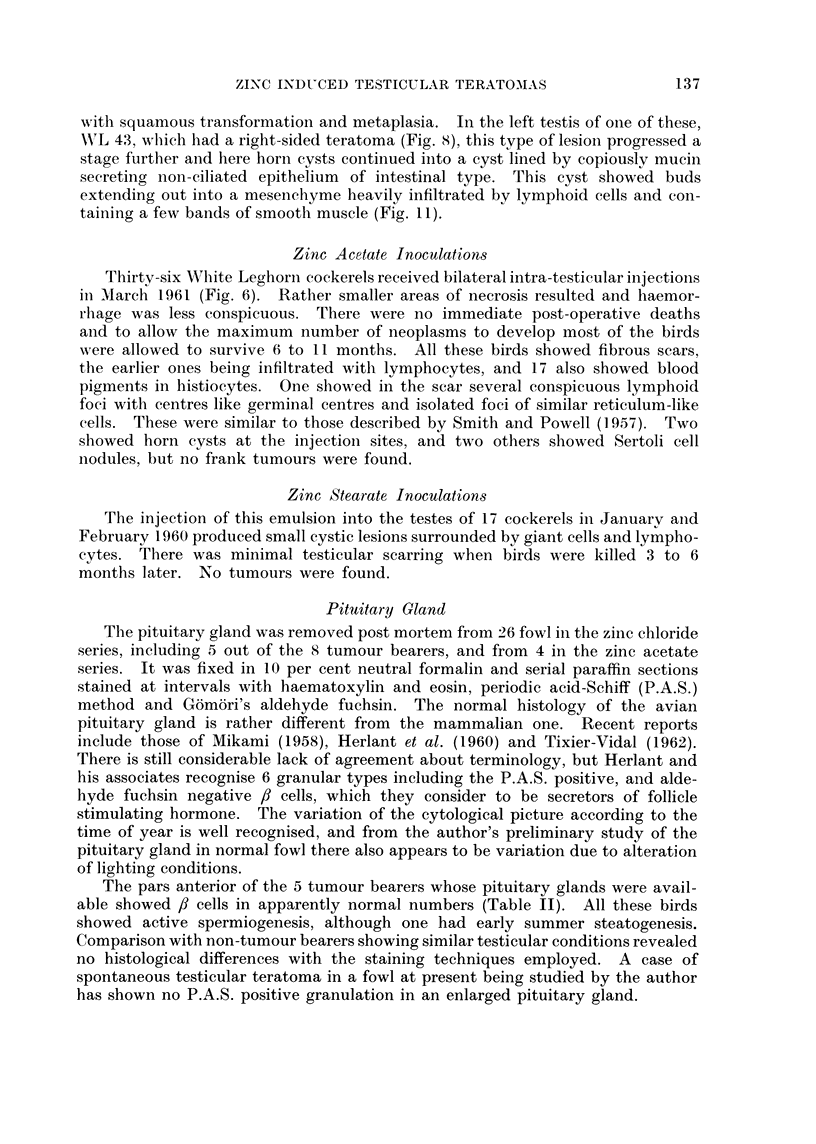

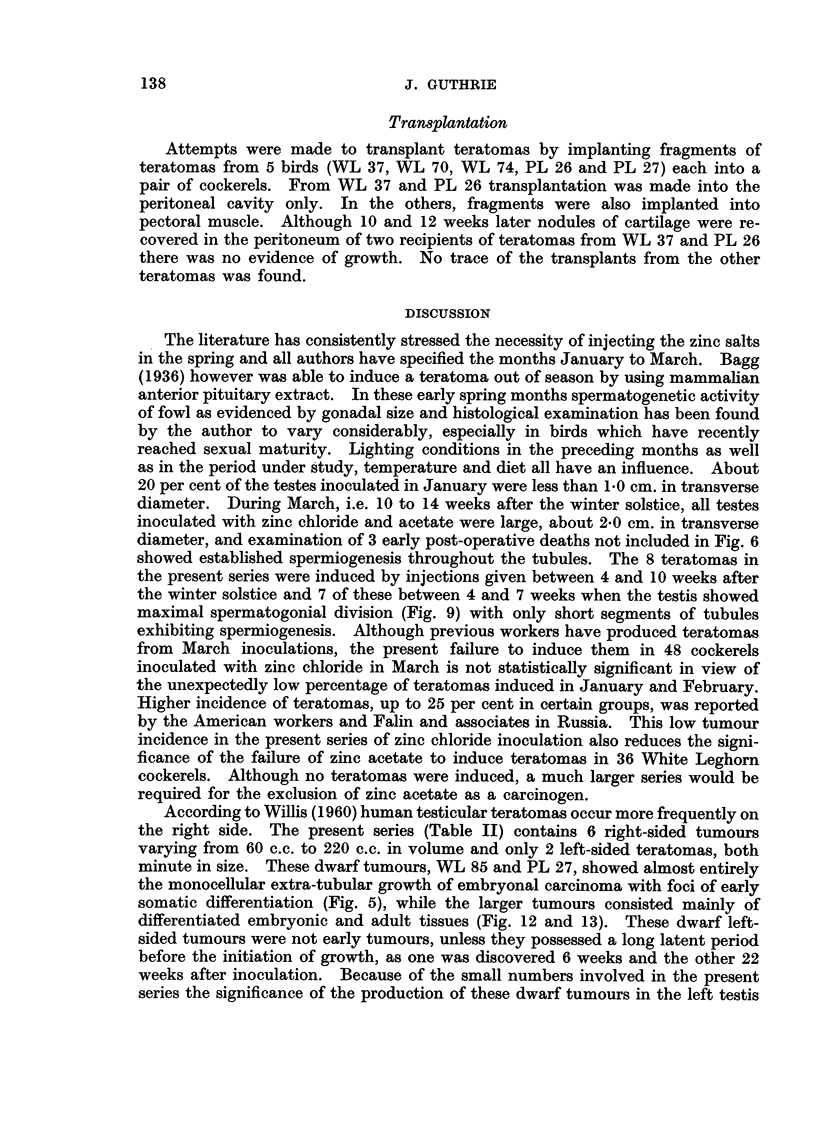

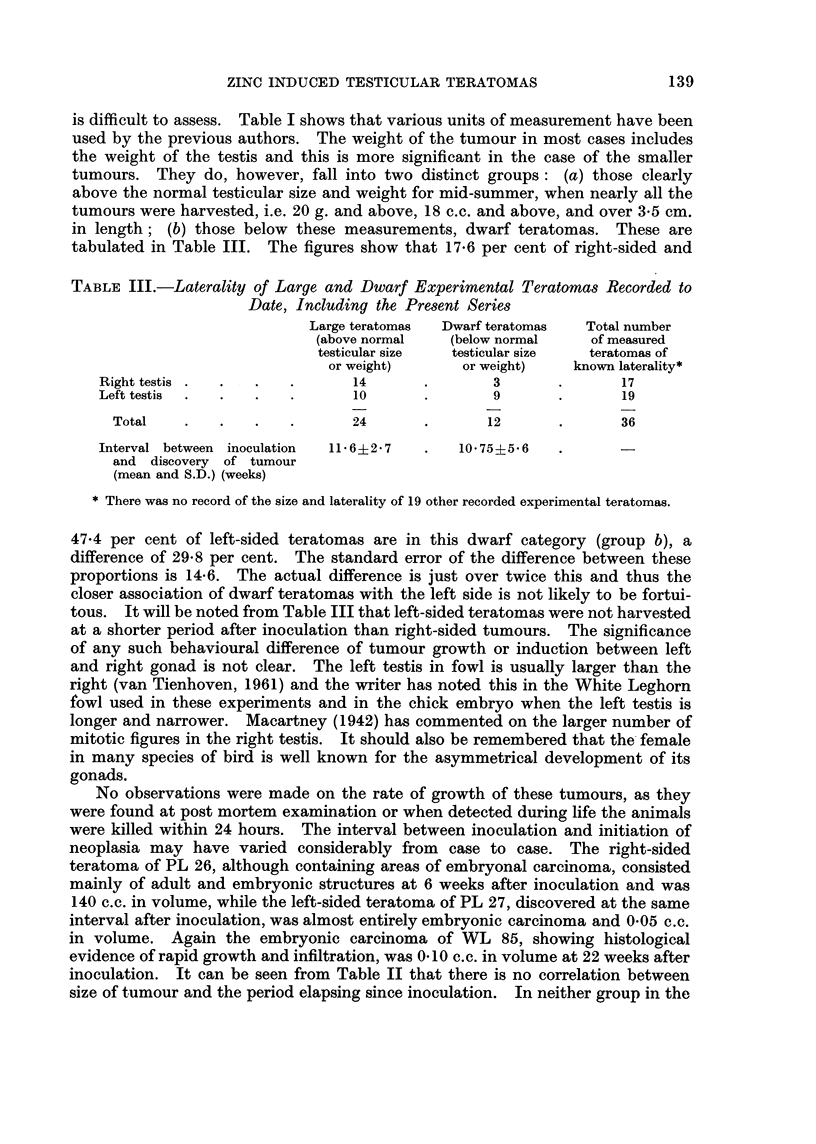

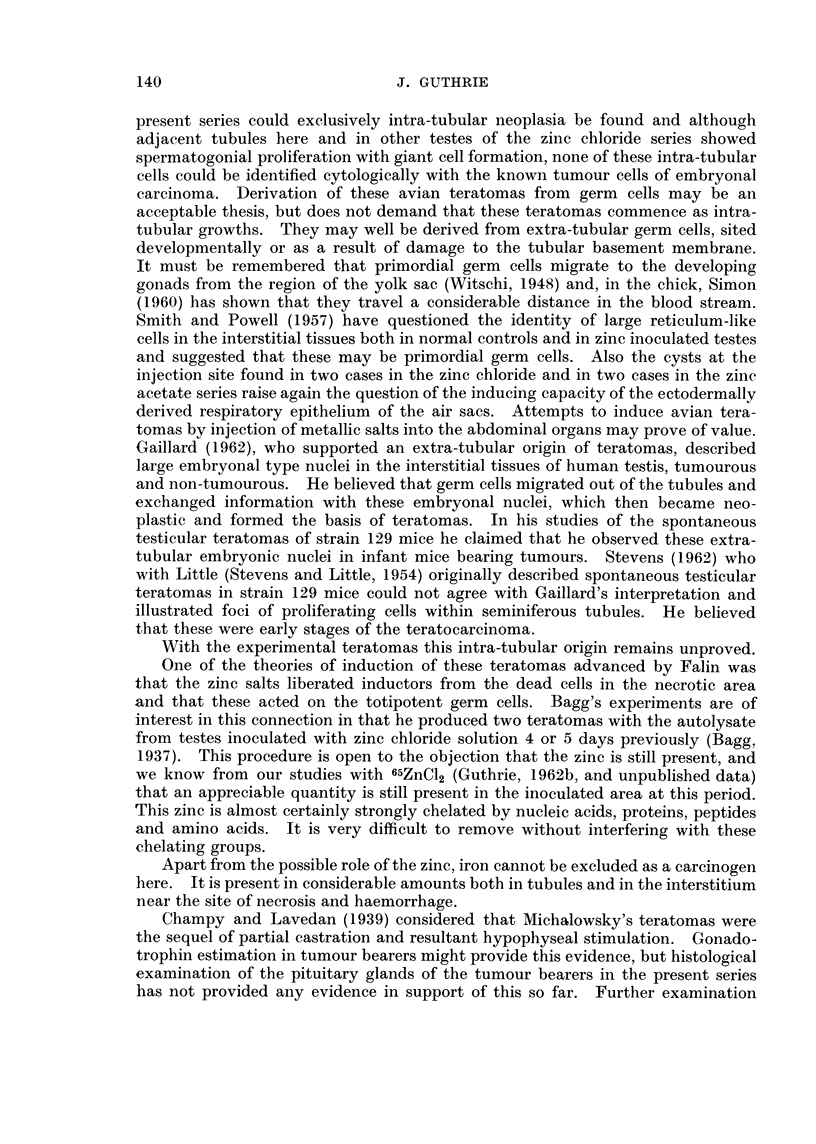

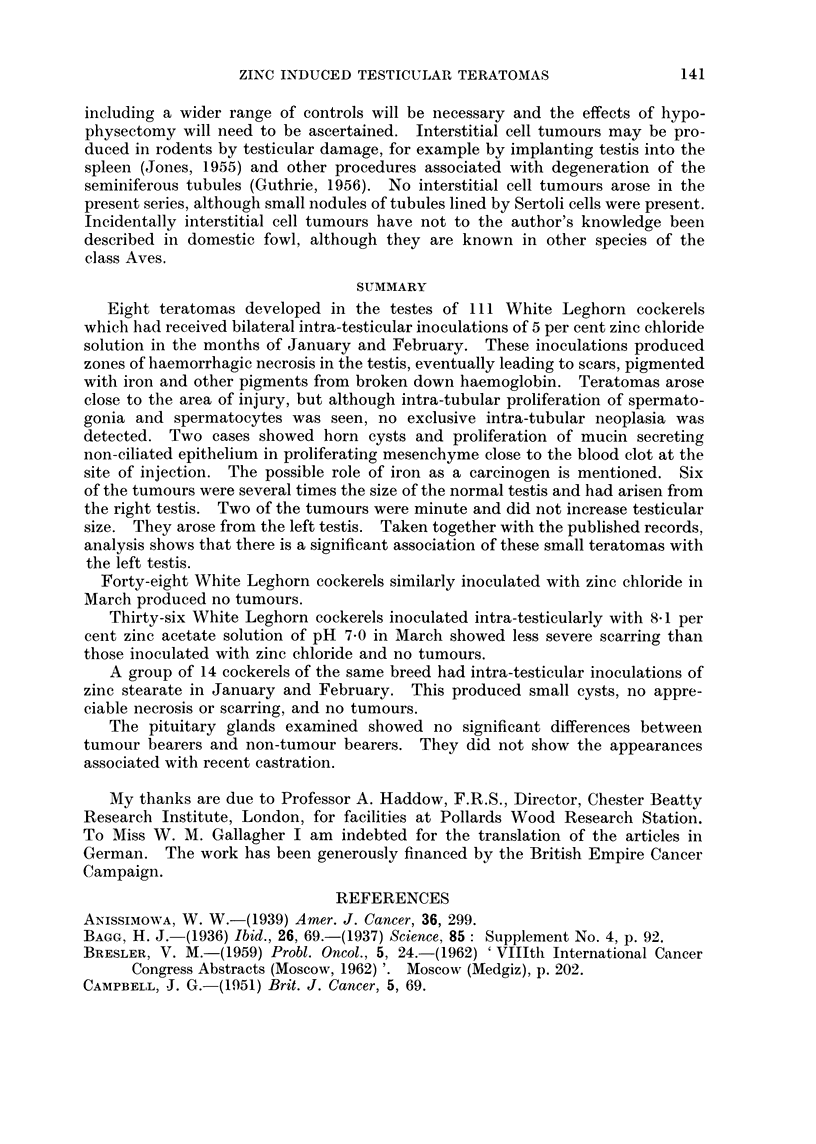

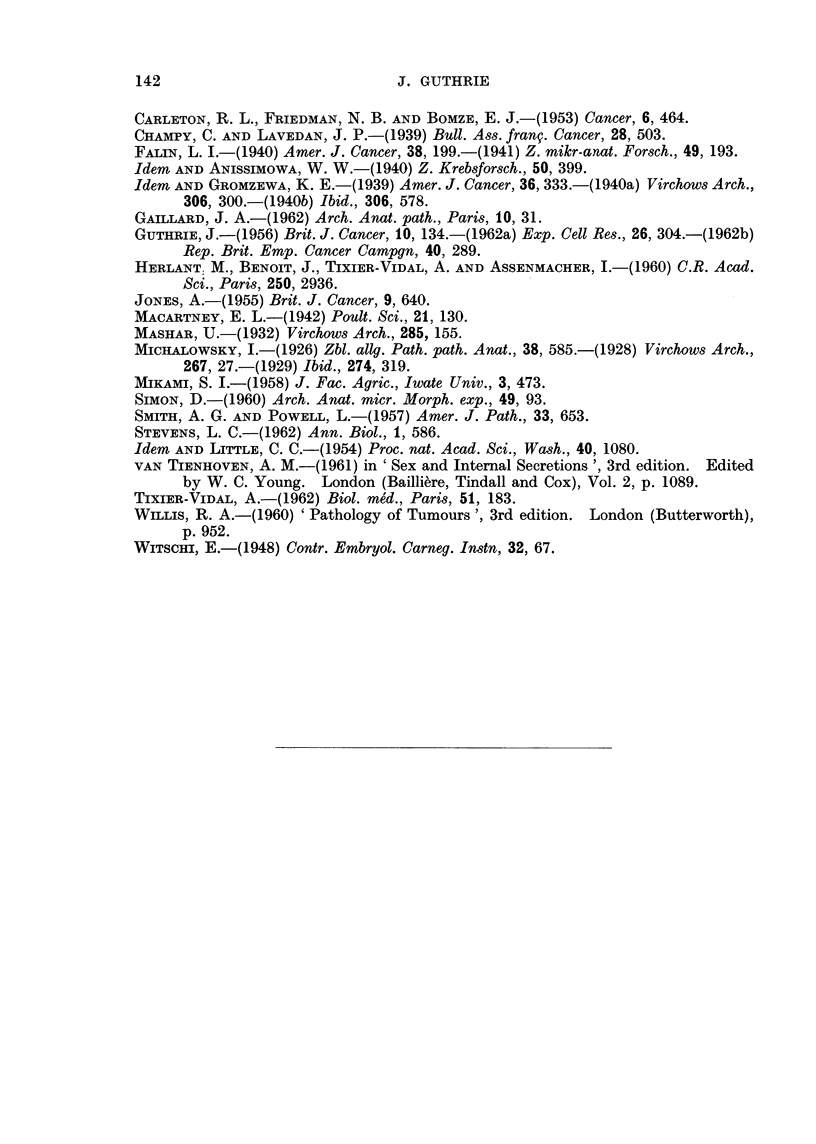


## References

[OCR_01093] CARLETON R. L., FRIEDMAN N. B., BOMZE E. J. (1953). Experimental teratomas of the testis.. Cancer.

[OCR_01109] HERLANT M., BENOIT J., TIXIER-VIDAL A., ASSENMACHER I. (1960). [Hypophysial modifications during the annual cycle in the Peking drake].. C R Hebd Seances Acad Sci.

[OCR_01133] TIXIER-VIDAL A. (1962). [Cytology of the anterior lobe of the adenohypophysis of birds].. Biol Med (Paris).

